# Immune System Disorders, Cancer and Viral Infections: A New Treatment Opportunity for the Immune Checkpoint Inhibitors

**DOI:** 10.3390/life11121400

**Published:** 2021-12-15

**Authors:** Alejandro Olivares-Hernández, Luis Figuero-Pérez, José Pablo Miramontes-González, Álvaro López-Gutiérrez, Rogelio González-Sarmiento, Juan Jesús Cruz-Hernández, Emilio Fonseca-Sánchez

**Affiliations:** 1 Department of Medical Oncology, University Hospital of Salamanca, 37007 Salamanca, Spain; figuero44@gmail.com (L.F.-P.); varolopez94@hotmail.com (Á.L.-G.); efonseca@saludcastillayleon.es (E.F.-S.); 2 Institute for Biomedical Research of Salamanca (IBSAL), University of Salamanca, 37007 Salamanca, Spain; gonzalez@usal.es (R.G.-S.); jjcruz@usal.es (J.J.C.-H.); 3 Department of Internal Medicine, University Hospital Rio Hortega, 47012 Valladolid, Spain; 4 Faculty of Medicine, University of Valladolid, 45005 Valladolid, Spain; 5 Faculty of Medicine, University of Salamanca, 37007 Salamanca, Spain

**Keywords:** viral infections, immunotherapy, immune checkpoint inhibitors, solid tumours, survival

## Abstract

The relationship between viral infections and cancer is well known and has been established for decades. Multiple tumours are generated from alterations secondary to viral infections 2 resulting from a dysregulation of the immune system in many cases. Certain causal relationships, such as that between the Epstein–Barr virus (EBV) in nasopharyngeal cancer or hepatitis C and B viruses in hepatocarcinoma, have been clearly established, and their implications for the prognosis and treatment of solid tumours are currently unknown. Multiple studies have evaluated the role that these infections may have in the treatment of solid tumours using immunotherapy. A possible relationship between viral infections and an increased response to immune checkpoint inhibitors (ICIs) has been established at a theoretical level in solid neoplasms, such as EBV-positive cavum cancer and human papillomavirus (HPV)-positive and oropharyngeal cancer. These could yield a greater response associated with the activation of the immune system secondary to viral infection, the consequence of which is an increase in survival in these patients. That is why the objective of this review is to assess the different studies or clinical trials carried out in patients with solid tumours secondary to viral infections and their relationship to the response to ICIs.

## 1. Background

Infections are one of the leading preventable causes of cancer in the world, as many are involved in the development of different tumours. The primary microorganisms that are involved in cancer are found in Table 1, and viral infections have a special importance due to their possible involvement in the treatments that can be prescribed [1]. Multiple viruses have been linked to cancer, of which human papillomavirus (HPV), Epstein–Barr virus (EBV), and hepatitis B and C viruses (HBV and HCV) have been the most studied [2].

Approximately 10% of cancers worldwide can be attributed to viral infections [3]. The three tumours that have the greatest importance in this aspect are as follows: (1) Hepatocarcinoma (HCC) is the sixth neoplasm with the highest incidence worldwide, is third in mortality and is caused by endemic infection by HBV and HCV in African countries; (2) Cervical cancer, whose pathogenesis is attributable in most cases to HPV, is ninth in mortality worldwide and (3) Nasopharyngeal cancer, although not a common tumour (less than 5% of cancer cases worldwide), in certain countries, the incidence reaches 20% due to endemic EBV infection, as is the case in the Cantonese population in China [4].

The causal mechanism by which these viral infections produce tumours is the continuous expression of oncogenic viral genes. These genes are responsible for regulating mechanisms of proliferation and cell death through which alteration can induce the processes of carcinogenesis. Among these examples are the oncoproteins E6 and E7 in HPV or latent membrane protein 1 (LMP1) in EBV. There are other mechanisms through which indirect carcinogenesis can be induced. In these cases, the tumours originate through continuous tissue damage alongside consequent tissue regeneration and chronic inflammation [5]. The most paradigmatic case is HCC with cirrhosis caused by HBV and HCV [6]. Other infections, such as human immunodeficiency virus (HIV), are not carcinogenic, but alter the functions of the immune system, causing an increase in tumour evasion and the incidence of associated tumours [7].

The alterations produced by these infections in the cell cycle, and consequently in the immune system, could influence the response to immunotherapy treatments, especially in treatments that use immune checkpoint inhibitors (ICIs) [8]. These drugs can induce an increase in the response of T lymphocytes by activating and inhibiting different receptors associated with the immune response [9]. Depending on the viral status of the tumours (positivity or negativity in its pathogenesis) or the viral load, the response to these ICIs in the future could be predicted from these viruses’ associations with the mechanisms of antitumor immunity. Therefore, the objective of this review is to assess the current scientific evidence in the different studies that used immunotherapy in tumours secondary to viruses. The predictive value of these infections in their response to ICIs may be key to the performance of precision medicine in these patients in the future.

## 2. Immune Checkpoint Inhibitors

The first works linking the immune system to antitumour activity can be traced back to the late nineteenth century. In 1891, William Coley first injected bacteria into a tumour, thereby reducing the tumour size in a patient affected by sarcoma [10]. The histological analysis of human tumours has revealed the presence of very heterogeneous immune infiltrates across different tumours and patients [11]. These infiltrates include different subpopulations of T cells and cells with innate immunity. Their existence has enabled the development of oncological therapies that are based on the modulation of the immune system [12].

Within the variety of treatments for solid tumours, the most important mechanism of modulation is the negative co-stimulation pathway of the tumour microenvironment. The inhibition of T cells by tumour cells occurs through two main inhibitory pathways: the cytotoxic T-lymphocyte antigen 4 (CTLA-4) protein receptor and programmed cell death protein 1 (PD-1) [13,14]. These two are the pathways through which ICIs act by negatively regulating these receptors and consequently stimulating T lymphocytes. The main drugs that target these receptors are found in Table 2.

One of the biggest current problems that ICIs have is the lack of knowledge about which patients will respond favourably to these treatments. The best-known biomarker to date is PDL-1, which, assessed by immunohistochemistry, makes it possible to predict the response to ICIs, although it is far from perfect. Other biomarkers, such as ECOG (Eastern Cooperative Oncology Group), tumour-infiltrating lymphocytes (TILs) and lactate dehydrogenase, are also being studied as possible biomarkers. Among them are viral infections, which may have a promising applicability in the future [15,16].

One of the most well-known risk factors for cancer is viral infections such as HPV, EBV or viruses related to viral hepatitis. It is unknown how these infections influence treatments with ICIs for the different tumours, especially in solid neoplasms where immunotherapy has a more important role. To date, the studies have yielded contradictory results; no studies have evaluated this association as their main objective. At a theoretical level, these infections would lead to greater activation of the immune system with a greater mobilisation of T lymphocytes to decrease viral activity and slow down carcinogenesis. Therefore, increasing the action of these T lymphocytes using ICIs will cause greater destruction of tumour cells than in basal conditions where, in many cases, tumour cell immune evasion is very high [8]. As indicated in the Introduction, this study studies the current evidence in this field to discover the role of these infections in the response to immunotherapy.

## 3. Human Papillomavirus Infection

Currently, HPV is a determining factor in the genesis of different tumours, among which are head and neck (especially the oropharynx), cervix, vulva, anus, and other cancers (Table 1) [17]. More than 100 genotypes are known and have been classified according to their risk of oncological pathogenesis. The best-known viruses to date are HPV subtypes 16, 18, 31 and 33 [18]. Different vaccines have been developed to prevent carcinogenesis, including for various subtypes of HPV [19].

The involvement of HPV in carcinogenesis is mainly due to the presence of two viral oncogenes, E6 and E7. These genes encode proteins that modify the cell cycle, producing the oncological transformation of cells. The E6 protein encodes 151 amino acids with a molecular weight of 16–18 kDa [20]. This protein leads to the blocking of apoptosis by degrading p53 with the consequent increase in mutations in cellular DNA. However, the E7 protein encodes 98 amino acids with a molecular weight of 10 kDa. This protein acts on tumour suppressor proteins of the retinoblastoma family, which in turn interact with transcription factors of the E2F family [21]. These alterations prevent correct cell replication, facilitating the processes of carcinogenesis.

Tumours caused by HPV have different characteristics compared to similar tumours not resulting from infection. The most paradigmatic case of this can be observed in oropharyngeal cancer, where HPV infection causes up to 70% of cases [22]. HPV-positive tumours are larger lesions with greater nodal involvement than those with HPV-negative. The older they are, the greater their response to chemotherapy and radiotherapy treatments, creating a more favourable prognosis than HPV-negative tumours. For all these reasons, the TNM classification of HPV-positive oropharyngeal tumours is different from HPV. The immune system plays a key role in the development of all cancers, especially those caused by HPV infections [23]. The immunological escape performed by tumours is produced by different mechanisms in cases of HPV-positive tumours compared to conventional tumours. In the case of infected patients, the deficient immune response presented by these patients contributes to the maintenance of the virus. HPV found in epithelial cells can inhibit antitumor response pathways via the immune system through reduced protein translation of key sequences for the response to tumour cells. It has been seen in different studies that the E6 protein is involved in low-grade lesions and the oncoprotein E7 in high-grade lesions.

One of the main tumours where the association between HPV infection and response to immunotherapy has been studied is head and neck cancer. In these tumours, HPV infection leads to a better prognosis from a better response to chemotherapy treatments, especially in the oropharynx, where its involvement has been more studied [24]. However, its role in responding to ICIs is more unknown, as is the involvement of HPV. Current clinical guidelines in head and neck cancer indicate that therapeutic decisions depend not on the value of PDL-1 but on the combined positive score (CPS), which assesses the expression of PDL-1 together in tumours and immune cells. As such, the treatment of head and neck tumours in the metastatic or advanced stages is based on ICIs in monotherapy if the CPS ≥ 20 and chemotherapy plus immunotherapy in cases of CPS < 20. Therefore, it is important to study whether HPV status influences the response to immunotherapy to adapt these clinical guidelines to HPV status. This would be important in cases of CPS ≥ 20, where treatment should combine ICIs with chemotherapy, or CPS < 20, where an immunotherapy treatment might be sufficient to avoid the toxicity of chemotherapy [25,26].

HPV tumour positivity has been linked to a better response to immunotherapy and a higher percentage of response. In the KEYNOTE-012 study, which evaluated the safety and clinical activity of pembrolizumab in the treatment of metastatic or recurrent head and neck cancer, a tendency towards greater response and survival was observed in HPV-positive versus HPV-negative oropharyngeal tumours [27]. Another study (HAWK study) evaluating the efficacy of durvalumab in metastatic or recurrent head and neck cancer that had progressed to platinum analysed the results based on HPV status. This study also observed through an ad hoc analysis how survival and the percentage of responses were higher in patients positive for HIV [28]. However, despite the above results, a systematic review by Patel et al. did not show that there was a better response in HPV-positive tumours. In this review, there were no statistically significant differences (*p* = 0.06) in the overall response rate (ORR) [29]. Therefore, currently, the data on the head and neck are contradictory, although the data are encouraging, and it is possible that HPV status is a predictive biomarker of response to immunotherapy in head and neck tumours.

Along with the above tumours, the cancers most related to HPV are those of the gynaecological sphere. In this case, the influence of HPV on the response to immunotherapy has been less studied than that in head and neck tumours, although there are important data in this regard, especially in cervical tumours. An interesting study in this regard is CheckMate-358, which was evaluated in a phase 1/2 clinical trial that treated patients with HPV-associated gynaecological tumours with nivolumab [30]. The main objective of the study was to evaluate ORR in these tumours. The disease control rate was 70.8%, with good ORR rates in these HPV-related tumours. Although few studies have subsequently evaluated this association, it opens a way to use these viruses as a biomarker of response to ICIs in gynaecological tumours.

Several studies have examined whether PDL-1 expression in these tumours is related to HPV infection. Studies by Mezache et al. and Liu et al. showed that there was a positive correlation between the two. PDL-1 expression was also associated with an increased likelihood of metastasis, progression, and worse prognosis in cervical tumours [31,32]. Therefore, there could be a relationship between HPV positivity and a worse prognosis for cervical tumours whose nexus would be the expression of PDL-1. This is true not only for already established tumours, but also in precursor lesions. The study conducted by Usta et al. showed how the expression of PDL-1 is higher when the degree of dysplasia of cervical epithelial cells is increased, and this expression is related to the presence of HPV infection [33].

These results demonstrate the possibility of a new biomarker for predicting response to immunotherapy, such as viral load and HPV infection in gynaecological tumours. The relationship between infection and the increased expression of PDL-1, and in turn, with worse outcomes in these tumours, highlights the possibility that it will be necessary to adapt the treatments for these patients to the expression of PDL-1 and the status of HPV in the future.

## 4. Epstein-Barr Virus

EBV is the primary agent of infectious mononucleosis, persists asymptomatically for life in nearly all adults and is associated with the development of B cell lymphomas, T cell lymphomas, Hodgkin lymphoma, nasopharyngeal carcinoma (NPC) and gastric carcinomas (GC) in certain patients. The oncogenic properties of EBV have been studied for a long time [34]. The primary neoplasms associated with EBV are lymphomas, NPC, and GC, reflecting the primary cellular targets of viral infection in vivo, specifically B cells and tonsillar epithelium, respectively. The virus utilises multiple mechanisms to promote neoplasm, including the activation of the B cell growth program, immune evasion and inactivation of tumour suppressors [35,36].

**Figure 1:**
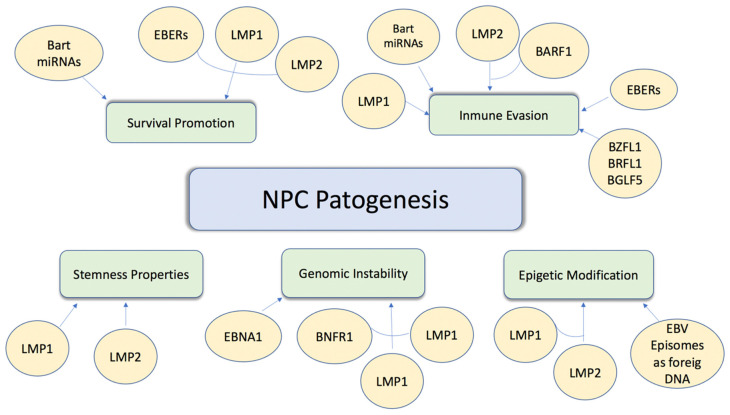
Carcinogenesis induced by EBV infection. Molecular mechanisms through which EBV induces proliferation, absence of cell death, and increased cell survival.

EBV-associated NPC is one of the most common head and neck malignancies, and unfortunately, 70% of NPC patients have locally advanced disease upon initial diagnosis. A large body of evidence supports the role of EBV as a primary etiologic agent in the pathogenesis of nasopharyngeal carcinoma [37]. This includes the detection of both EBV DNA and EBV gene expression in precursor lesions and tumour cells. NPC cells express a specific subgroup of EBV-latent proteins, including Epstein–Barr virus nuclear antigen 1 (EBNA1) and two integral membrane proteins, latent membrane proteins 1 and 2 (LMP1 and LMP2). The establishment of stable infection from EBV in pre-invasive nasopharyngeal epithelium represents an early stage of NPC development. Details of the potential involvement of EBV infection in NPC development are summarised below in Figure 1 [38].

Treatment of early and locally advanced NPC is based on radiotherapy and chemotherapy, depending on the stage. However, ICIs appear to be a promising approach for the treatment of EBV-associated advanced NPC [39]. Different studies have assessed whether the positivity of EBV infection in these tumours influences the response to immunotherapy. A multinational study (NCI-9742) evaluated the antitumor activity of nivolumab in NPC [40]. In this study, patients with recurrent or metastatic (R/M) NPC who were treated with nivolumab until disease progression and plasma-based biomarkers were studied. A total of 44 patients were evaluated, and the ORR was 20.5%. There was no statistical correlation between ORR and plasma EBV DNA clearance. Even so, the promising result of nivolumab in R/M NPC has driven interest in exploring the use of ICIs in EBV-associated NPC [41].

In a phase II trial (POLARIS-02) of 190 patients with treatment-refractory disease treated with toripalimab, the ORR was 21% [41]. In this study, a reduction of ≥50% in the plasma DNA copy number of EBV at day 28 of treatment was associated with a statistically significantly improved ORR. These results showed a possible association between the presence of EBV infection and a poor response to ICIs. Therefore, in these patients, it would be essential to intensify the treatment to adapt it to the patient’s needs.

Other tumours with an important association with EBV infection are gastric tumours. It has been estimated that between 5 and 10% of gastric cancers worldwide are associated with EBV [42], although the role of EBV in gastric carcinogenesis, either directly or as a secondary effect, has been debated [43]. EBV-associated gastric cancers (EBV-GC) have distinct clinicopathologic characteristics, including male predominance, preferential location in the gastric cardia or postsurgical gastric stump, lymphocytic infiltration, a lower frequency of lymph node metastasis, perhaps a more favourable prognosis and a diffuse type of histology in most series [44,45]. In addition, in part due to the overexpression/amplification of programmed cell death ligand 1 (PDL-1) in EBV-GC, these tumours are good candidates for therapy with immune checkpoint inhibitors.

These tumours present a similar behaviour and molecular characteristics to gastric tumours that present microsatellite instability. For this reason, treatment with ICIs is currently a great opportunity for this type of EBV-positive tumour. In the study carried out by Xie et al., EBV was shown to be a promising biomarker for gastric tumours, where there is a probable increase in ORR in EBV-positive tumours treated with immunotherapy [46]. This could play a key role in the future when immunotherapy can be standardised as a first-line treatment for EBV-positive gastric adenocarcinoma.

The last of the EBV-related tumours that demonstrate a relationship between the response to ICIs and the virus is lymphomas. High PD-L1 expression has been associated with a range of EBV-positive lymphomas. In a study by Kim et al., the efficacy of pembrolizumab was analysed in patients with relapsed or refractory non-Hodgkin lymphomas based on the presence or absence of EBV. The results showed that a high expression of PDL-1 was associated with EBV-positive tumours. Along with the above, the efficacy of pembrolizumab in these EBV-positive tumours was higher than in EBV-negative tumours. On the contrary, in EBV tumours with low PDL-1 expression, the response to pembrolizumab was poor, and its efficacy was low [47].

This study shows how EBV-positive lymphomas have a better response to immunotherapy. Although clinical trials are needed to study the influence of EBV status on the response to immunotherapy, there is a possibility of direct immunotherapy treatments for EBV-positive non-Hodgkin lymphomas.

## 5. Hepatitis B and C Viruses

Chronic infections due to HBV and HCV are estimated to be responsible for almost three-quarters (73.4%) of HCC in the world [48]. The geographic variability of the incidence of HCC and its heterogeneity has been widely associated with the different distributions of HBV and HCV infections worldwide. Globally, HBV accounts for about 80% of virus-associated HCC cases, especially in Africa and East Asia, the areas with the highest incidence HCC, while HCV infection, involved in about 20% of total HCC cases, seems to be mainly related to HCC development in low-incidence HCC areas like Western Europe and North America [49].

Carcinogenesis induced by HBV is a complex process in which integration of the viral DNA into host DNA at multiple sites is thought to be a crucial step. The evidence available demonstrates that HBV, both by synthesising some of its own proteins (HBx, PreS2/S and HBSP) and by inducing genetic alterations, can deregulate liver cell growth, proliferation, differentiation, repair mechanisms and apoptosis. The HBx protein plays a crucial role in liver oncogenesis as a co-factor or tumour promoter through its pleotropic functions [50,51]. HCV is a small, enveloped RNA virus belonging to the family Flaviviridae. HCV-induced HCC development is a multi-step process that involves the establishment of chronic HCV infection, chronic hepatic inflammation, progressive liver fibrosis, initiation of neoplastic clones accompanied by irreversible somatic genetic/epigenetic alterations and progression of the malignant clones in a carcinogenic tissue microenvironment [52].

Advanced HCC is the most frequent liver cancer, and immunotherapy has been explored to improve survival outcomes. Nowadays, scientific research is focusing especially on ICIs, anti-PD1, anti-PD-L1 and anti-CTLA4 monoclonal antibodies, as single agents or in combination with other immunotherapy agents, target therapies, anti-vascular endothelial growth factors and other agents targeting specific molecular pathways. The role that viral infection may have in the response to ICIs is to be discovered, despite several significant studies [53,54].

In a phase 1/2 trial with nivolumab (CheckMate 040) in patients with advanced HCC, the ORR was 20% in HCV-infected patients and 14% in HBV-infected patients. Disease control was achieved in 66% of patients infected with HCV and 55% of patients infected with HBV. Six-month overall survival (OS) was 85% (95% confidence interval (CI): 72–93) in the HCV-infected cohort and 84% (95% CI: 71–92) in the HBV-infected cohort [55]. In another study’s phase 3 trial with nivolumab versus sorafenib (CheckMate 459) in patients with advanced HCC, no statistically significant differences were observed between treatment subgroups based on the presence or absence of infection with HBV or HCV viruses [56].

In another study, pembrolizumab was evaluated in a phase 2 clinical trial where its efficacy in HCC after progression to sorafenib (KEYNOTE-224) was studied. In this study, no differences were observed in response and survival between patients depending on whether they were infected with the HBV or HCV viruses. This study was followed by another phase 3 trial (KEYNOTE-240) in which the efficacy of pembrolizumab versus best supportive care was compared in HCC patients who had progressed to sorafenib. In this study, despite not achieving the primary endpoint of OS and PSF, the analysis by subgroups showed that OS was favourable for patients with HBV infection versus HCV or uninfected patients. Similarly, progression free survival (PFS) was better in those patients with HCV infection compared to the other two subgroups [57].

The most important study to date with ICIs in HCC is the IMbrave150 trial [58]. In this study, the first-line efficacy of the combination of atezolizumab plus bevacizumab versus sorafenib was evaluated as the first-line treatment for patients with HCC. In this study, the primary objectives of OS and PFS were achieved in all treatment subgroups (including those based on the presence of viral infection). Given the above studies, it is likely that the efficacy of ICIs is independent of the presence of HBV or HCV infection. In certain patients, it is possible that infection is a predictor of response to ICIs; however, we do not know what characteristics these patients have. The KEYNOTE-240 study showed how, in certain cases, infection is predictive of response to ICIs, although clinical trials are needed to test this hypothesis.

Published clinical trials evaluating the efficacy of ICIs in cancers associated to viruses are summarised in Table 3.

## 6. Other Viruses

Of the other viruses involved in the carcinogenesis of different tumours, the most studied as a predictor of response to ICI is the Merkel cell polyomavirus (MCPyV). To date, two studies have approved immunotherapy for Merkel cell carcinoma. The first of these was JAVELIN Merkel 200 [59]. In this study, the efficacy of avelumab in Merkel cell carcinoma was evaluated in 88 patients. The trial showed how avelumab presented prolonged responses in these tumours without differences between the different subgroups studied. In this case, there were no differences in response depending on whether the tumours were caused by the virus or not.

The other study was NCT02267603, in which the efficacy of pembrolizumab in treating Merkel cell carcinoma was evaluated. In this study, the ORR was 56%, not observing differences in response between the different analysis subgroups. PDL-1 expression was higher in tumours originated by the virus; however, PDL-1 values did not influence the response that was independent of the presence of the virus. Therefore, to date, it has not been shown that infection by the MCPyV virus is a predictor of response to ICIs [60].

Regarding other oncogenic viruses, such as human T-lymphotropic virus 1 (HTLV-1) or human gammaherpesvirus 8 (HHV-8), given the limited information on immunotherapy’s effects in these tumours, there are no data that have reported on the influence of the viruses on the response to immunotherapy. Future studies in this area will be difficult to conduct due to the low prevalence of these tumours and the difficulty of conducting studies with ICIs in these tumours.

## 7. Conclusions

Immunotherapy currently represents a largely unknown field of research. Learning about the response mechanisms to ICIs and the biomarkers that represent them constitutes one of the challenges of oncology for the realisation of precision medicine. The available data on the influence of viruses on the response to immunotherapy are scarce, although it seems important in tumours, such as those of the head and neck and those related to EBV. Questions such as whether the influence is limited to the presence of the virus or whether the viral load is important in tumours where it is key in carcinogenesis, such as HCCs, remain to be answered. However, it is likely that in the not-too-distant future, we will know if these tumours secondary to various viruses will benefit from personalised medicine, where immunotherapy plays a key role in many cases in monotherapy.

## Figures and Tables

**Table 1:** Viral infections and cancer. Causal relationship between infections and cancer.

Virus	Cancer	Prevalence in the Tumour
HPV	Cervix	100%
	Penile	50%
	Vaginal	70%
	Anal	80–90%
	Vulvar	40–50%
	Oropharynx	20–50%
HBV	Liver	20–60%
HCV	Liver	20–30%
EBV	Hodgkin’s lymphoma	40–90%
	Burkitt’s lymphoma	20–100%
	Nasopharyngeal carcinoma	50–100%
MCPyV	Merkel cell carcinoma	50–80%
HHV-8 (KSHV)	Kaposi´s sarcoma	100%
HTLV-1	Adult T-cell leukaemia and lymphoma	100%

**Abbreviations:** Human papillomavirus infection (HPV); Hepatitis B virus (HBV); Hepatitis C virus (HCV); Epstein–Barr virus (EBV); Merkel cell polyomavirus (MCPyV); Human gammaherpesvirus 8 (HHV-8); Kaposi’s sarcoma-associated herpesvirus (KSHV); Human T-lymphotropic virus 1 (HTLV-1).

**Table 2:** ICIs currently approved by the European Medicines Agency (EMA) in the treatment of solid tumours. Different examples of your current indications are shown in the right column.

Immune Checkpoint Inhibitors	Immunoglobulin Type	Target Molecule	Treatment of Different Tumours
Ipilimumab (MDX-010)	IgG-1κ	CTLA-4	Advanced melanoma Advanced renal cancer
Pembrolizumab (MK-3475)	IgG-4κ	PD-1	Advanced melanoma and adjuvant Metastatic non-small cell lung cancer Advanced bladder cancer Advanced head and neck cancer
Nivolumab (MDX-1106)	IgG4	PD-1	Advanced melanoma and adjuvant Metastatic non-small cell lung cancer Advanced bladder cancer Advanced head and neck cancer Advanced renal cancer
Cemiplimab (L01XC33)	IgG4	PD-1	Cutaneous Squamous Cell Carcinoma
Atezolizumab (MPDL3280A)	IgG1	PD-L1	Advanced bladder cancer Metastatic non-small cell lung cancer
Durvalumab (MEDI4736)	IgG1	PD-L1	Locally advanced unresectable non-small cell lung cancer
Avelumab (MSB0010718C)	IgG1	PD-L1	Metastatic Merkel cell carcinoma

**Table 3:** Main studies evaluating the efficacy of ICIs in tumors secondary to viral infections that evaluate viruses as a predictive biomarker of response to immunotherapy.

Clinical Trial	Phase	Tumours	Drugs	Relationship between ICIs and Viruses
KEYNOTE-012	1b	Squamous cell carcinoma of head and neck	Pembrolizumab	Tendency to greater response and survival was observed in HPV+ versus HPV- oropharyngeal tumours.
HAWK	2	Squamous cell carcinoma of head and neck	Durvalumab	In an ad hoc analysis the percentage of responses was higher in patients with HPV+.
CheckMate-358	1/2	Recurrent or metastatic cervical, vaginal, or vulvar carcinoma	Nivolumab	Disease control rate in gynecological tumours VPH+ 70.8%.
NCI-9742	2	Recurrent and metastatic nasopharyngeal carcinoma	Nivolumab	No statistical correlation between ORR and plasma EBV DNA clearance
POLARIS-02	2	Recurrent or metastatic nasopharyngeal carcinoma	Toripalimab	A reduction of ≥50% in the plasma DNA copy number of EBV at day 28 of treatment was associated with a statistically significantly better ORR
Kim et al. (not clinical trial, prospective study)	-	Relapsed or refractory non-Hodgkin lymphomas	Pembrolizumab	Tendency a high expression of PDL-1 in EBV+ tumours.
CheckMate-040	1/2	Advanced hepatocellular carcinoma	Nivolumab	Better ORR and disease control in HCV infected versus HBV infected tumours.
CheckMate-459	3	Advanced hepatocellular carcinoma	Nivolumab	No differences by subgroups.
KEYNOTE-224	2	Advanced hepatocellular carcinoma	Pembrolizumab	No differences by subgroups.
KEYNOTE-240	3	Advanced hepatocellular carcinoma	Pembrolizumab	Better OS in HBV+ versus HCV- or not infected.
IMbrave 150	3	Advanced hepatocellular carcinoma	Atezolizumab	No differences by subgroups.
JAVELIN Merkel 200	2	Metastatic Merkel cell carcinoma	Avelumab	No differences by subgroups.
NCT02267603	2	Metastatic Merkel cell carcinoma	Pembrolizumab	High PDL-1 expression in MCPyV+ versus MCPyV- tumours. PDL-1 values did not influence in the response to Pembrolizumab.

## Data Availability

Not applicable.

## Abbreviations

CPS	combined positive score
CTLA-4	cytotoxic T-lymphocyte antigen 4
EBNA1	Epstein–Barr nuclear antigen 1
EBV	Epstein–Barr virus
EBV-GC	Epstein–Barr virus—gastric cancer
ECOG	Eastern Cooperative Oncology Group
GC	gastric cancer
HBV	hepatitis B virus
HCC	hepatocellular carcinoma
HCV	hepatitis C virus
HHV-8	human gammaherpesvirus 8
HIV	human immunodeficiency viruses
HPV	human papillomavirus
HTLV-1	human T-lymphotropic virus 1
ICIs	immune checkpoint inhibitors
LMP1	latent membrane protein 1
LMP2	latent membrane protein 2
NPC	nasopharyngeal carcinoma
MCPyV	Merkel cell polyomavirus
ORR	overall response rate
OS	overall survival
PD-1	programmed death 1
PDL-1	programmed death-ligand 1
PFS	progression free survival
R/M	recurrent/metastatic
TILs	tumour-infiltrating lymphocytes
